# PBMCs to Stress-Associated miR-18a-5p and miR-22-3p Ratios as New Indicators of Metabolic Syndrome

**DOI:** 10.1155/2020/8159342

**Published:** 2020-04-24

**Authors:** Yuxiang Huang, Liqin Zhao, Yuxiang Yan, Jingyu Chen, Pengfei Liu, Weicheng Xv, Ge Qian, Chijian Li, Shiyi Liang, Hequn Zou, Yongqiang Li

**Affiliations:** ^1^Department of Nephrology, Institute of Nephrology and Urology, The Third Affiliated Hospital of Southern Medical University, Guangzhou, China; ^2^Center of Health Management, The Third Affiliated Hospital of Southern Medical University, Guangzhou, China; ^3^Department of Epidemiology and Biostatistics, School of Public Health, Capital Medical University, Beijing, China; ^4^Department of Clinical Laboratory, The Third Affiliated Hospital of Southern Medical University, Guangzhou, China

## Abstract

**Purpose:**

Metabolic syndrome (MetS) is associated with chronic stress. miR-18a-5p and miR-22-3p are two miRNAs which can target the glucocorticoid receptor. This study looked at the changes in metabolic parameters and the predictive value of the peripheral blood mononuclear cells (PBMCs) to stress-associated miRNA ratios as new indicators in subjects with and without MetS in southern China. *Patients and Methods*. There were 81 participants (39 with MetS and 42 without MetS) in this cross-sectional study. The potential miRNAs were filtrated in the GEO database. The expression of miR-18a-5p and miR-22-3p in PBMCs was evaluated by quantitative reverse transcription polymerase chain reaction (qRT-PCR). The risk of miRNA and PBMCs to stress-associated miRNA ratios contributing to the presence of MetS was estimated by univariate and multivariate logistic regression models. The area under the receiver operating characteristic curve (AUC) was used to evaluate diagnostic accuracy.

**Results:**

MetS was positively correlated with cortisol, IL-6, lymphocyte to miR-18a-5p ratio (LT18R), lymphocyte to miR-22-3p ratio (LT22R), monocyte to miR-18a-5p ratio (MT18R), monocyte to miR-22-3p ratio (MT22R), PBMCs to miR-18a-5p ratio (PT18R), and PBMCs to miR-22-3p ratio (PT22R) and negatively associated with the expression levels of miR-18a-5p and miR-22-3p (*P* < 0.05). In addition, PT18R (odds ratio: 0.894; 95% CI: 0.823-0.966; *P* < 0.001) and PT22R (odds ratio: 0.809; 95% CI: 0.717-0.900; *P* < 0.001) were independent predictors of MetS, respectively. A receiver operating characteristic (ROC) curve analysis was performed to assess the value of the PT18R-PT22R (PMR) panel (odds ratio: 0.905; 95% CI: 0.838-0.971; *P* < 0.001) for predicting MetS. The area under the curve yielded a cut-off value of 0.608, with sensitivity of 74.4% and specificity of 95.2% (*P* < 0.001).

**Conclusion:**

In summary, miR-18a-5p and miR-22-3p in PBMCs may be important biomarkers of stress reaction and may play a role in vulnerability to MetS. Besides, the inflammatory cells to the two miRNA ratios demonstrated high accuracy in the diagnosis of MetS.

## 1. Introduction

MetS, once identified as “IR syndrome,” “syndrome X,” “hypertriglyceridemia waist,” and “the deadly quartet,” was firstly globally defined as MetS in 1998 by the Diabetes Consultation Group of the World Health Organization. MetS is a combination of several characteristics, including obesity, glucose intolerance, IR, dyslipidaemia, microalbuminuria, hypertension, nonalcoholic fatty liver disease (NAFLD), the proinflammatory state, and oxidative stress, resulting in an increasing risk of T2DM, cardiovascular disease (CVD), fatty liver, and cancer [1–3]. Five risk factors with different critical cut-offs are used to identify individuals with the MetS: waist circumference, circulating levels of triglycerides and high-density lipoprotein cholesterol (HDLC), fasting glucose, and blood pressure. IR also plays a pivotal role in the MetS [3, 4]. Among the various risk factors of MetS, chronic stress has now emerged as contributors to the development of MetS. Psychological stress can affect health through complex interactions among neuroendocrine responses and energy homoeostasis [5]. Glucocorticoid (GC) (cortisol in human beings) is the critical matter responding to stress, which can cause central obesity, hypertension, hyperlipidaemia, and glucose intolerance. GC action is mediated by the glucocorticoid receptor (GR), a nuclear receptor that regulates physiological events through activation or repression of target genes involved in inflammation, gluconeogenesis, and adipocyte differentiation [6].

In recent years, miRNAs have arisen global interest in complex processes in health and diseases, including MetS and its components, which are used as noninvasive biomarkers for diagnosis [7]. miRNAs are small (19–23 nucleotides), single-stranded noncoding RNA molecules involved in posttranscriptional control of genetic expression of a large number of genes, acting as regulators of mRNA degradation and/or blocking protein translation of distinctive parts of target proteins via binding to their corresponding sequences within the 3′-untranslated regions (3′UTRs) of target mRNAs [7, 8]. It has been manifested that miRNAs are stable in circulation because they are resistant to RNase's digestion. This shows great promise in becoming a novel diagnostic biomarker of MetS and its components [9]. Uchida and colleagues identified that miR-18a inhibited translation of GR mRNA in cultured neuronal cells and that high expression of miR-18a was present in Fischer 344 (F344) rats in the paraventricular nucleus [10]. Besides, using miRTarbase, we found that miR-22a-3p was predicted to target GR.

To our knowledge, few studies have investigated the expressions of miR-18a-5p and miR-22a-3p which can target GR in the MetS population. It has been suggested that the gene expression signature in PBMCs may provide an indicator of gene activation changes as differential response to stress in humans [11]. In this study, we analyzed the association between MetS and expression of miR-18a-5p and miR-22a-3p in PBMCs, which may unveil a new target for the prevention and treatment of stress-related disorders including MetS.

## 2. Methods

### 2.1. Subjects

This study was conducted among workers who took annual physical examination for at least three consecutive years at the health examination center of The Third Affiliated Hospital, Southern Medical University, from January to April 2019. A total of 39 diagnosed MetS (31M : 8F; mean age 40.38 ± 9.38 yrs.) cases were recruited. 42 health individuals without any components of MetS (19M : 23F; mean age 32.87 ± 7.40 yrs.) were selected (baseline characteristics are shown in Table 1). The diagnosis of MetS was in accordance with the common criteria of the International Diabetes Federation and the American Heart Association/National Heart, Lung, and Blood Institute [12]. Patients with acute or chronic infectious or immunological diseases, obvious liver and kidney dysfunction, severe heart diseases, pregnancy, mental illness or drug abuse, gastrointestinal diseases (such as chronic gastrointestinal disorders, diarrhoea, biliary tract infection, and enteritis), and serious diseases of the blood or the endocrine systems were excluded from the study. A structured questionnaire was used to collect information on demographic data, environmental exposure, and medical histories. Current cigarette smokers were defined as those who smoked ≥1 cigarette/day. Alcohol use was defined as the intake of wine/beer/cider/spirits ≥ 1 time per week. Physical activity was defined as walking or riding 15 min/day, doing sports or physical exercise > 2 h/week, or lifting or carrying heavy objects at work daily [13]. This study was approved by the hospital ethical committee, and informed consent was obtained from each participant.

### 2.2. Anthropometric Measurement

Anthropometric parameters including weight, height, waist circumference (WC), hip circumference (HC), and blood pressure were obtained using standard measurement. Body mass index (BMI) was calculated by dividing the weight (kg) by the squared value of height in metres.

### 2.3. Blood Sample Collection and RNA Extraction

Following an overnight fast, 5 ml venous blood sample from each subject was collected using EDTA anticoagulant tubes and processed within three hours. A three-milliliter sample was immediately centrifuged to retrieve plasma. PBMCs were isolated from 2 ml whole blood by Ficoll-Hypaque density gradient centrifugation. Immediately after, total RNA was extracted from PBMCs by the standard protocol of the TRIzol reagent (Invitrogen, New York, USA). The purity of RNA was determined using a BioPhotometer plus Eppendorf nucleic acid protein analyzer (Hamburg, Germany), and the integrity was evaluated using agarose gel electrophoresis stained with ethidium bromide. All RNA used had OD260/OD280 ratio > 1.8, and electrophoresis showed integrity was acceptable. The plasma and RNAs were then stored at -80°C until assayed.

### 2.4. Biochemical Analysis

Fasting plasma glucose (FPG), total cholesterol (TC), triglycerides (TG), and HDLC were measured using standard laboratory methods. Low-density lipoprotein cholesterol (LDLC) was calculated using the Friedewald formula. Insulin (INS) and IL-6 were calculated by the high-pressure liquid chromatography method. Plasma cortisol was measured by commercial radio immunoassays. Plasma TNF-*α* concentrations were evaluated by an enzyme-linked immunosorbent assay using a microplate reader. The intra-assay and interassay coefficients of variation were <5.5% and <10.0% for these assays, respectively. The degree of IR was determined using the HOMA-IR which was calculated using the following formula: [fasting insulin (mIU/l)∗fasting glucose (mmol/l)]/22.5.

### 2.5. miRNA Filtration

Gene Expression Omnibus (GEO), which is a database repository of high-throughput gene expression data and hybridization arrays, chips, and microarrays, was used to identify the differentially expressing miRNAs in PBMCs between the MetS group and healthy groups. The miRNA microarray data were collected from the GEO database, under the accession number GSE98896, which included the miRNA profiles of PBMC samples collected from 20 healthy children and 20 patients with the MetS group, as demonstrated on the previously published data [14]. The ∣Log Fold Change (logFC)∣(MetS group to control group) > 1.3 and *P* value < 0.05 were taken as cut-off values for detection of upregulated or downregulated miRNAs in the two groups. 60 miRNAs were detected. Among these miRNAs, we chose two miRNAs, miR-18a-5p and miR-22-3p, whose logFC was 1.42 and 1.41, respectively.

### 2.6. Quantitative Real-Time PCR

Quantification was performed with a two-step reaction process: reverse transcription (RT) and quantitative real-time PCR (qPCR). Each RT reaction consisted of 1 *μ*g RNA, 2.0 *μ*l of 10 mM dNTP (Promega), 0.5 *μ*l of RNase inhibitor (Promega), 0.5 *μ*l of universal primer (Qiagen), 0.5 *μ*l of miRNA-specific primer, 4 *μ*l of 5x buffer, 0.5 *μ*l of reverse transcriptase (Promega), and 12.0 *μ*l of nuclease-free water in a total volume of 20 *μ*l. U6 was used as the internal reference of miRNAs. Reactions were performed in an ABI PRISM® 7500 Sequence Detection System (Applied Biosystems, Foster City, USA) for 60 min at 42°C, followed by heat inactivation of RT for 10 min at 85°C. qPCR was performed with 20 *μ*l PCR reaction mixture that included 5 *μ*l of cDNA, 10 *μ*l of 2x SYBR Green qPCR SuperMix (Invitrogen), 0.5 *μ*l of forward primer (Qiagen), 0.5 *μ*l of reverse primer, and 4 *μ*l of nuclease-free water. Reactions were incubated in a 384-well optical plate at 95°C for 5 min., followed by 40 cycles at 95°C for 15 sec and 60°C for 32 sec. The specific generation of expected PCR product was confirmed by automated melting curve analysis. All samples were performed in triplicate (cDNA from the same PCR reaction but in separate wells). The expression levels of miRNAs were calculated using the 2^‐*ΔΔ*Ct^ method [ΔCt = mean Ct (miRNA of interest)‐mean Ct (U6), ΔΔCt = ΔCt(ΔCt of miRNA of interest in samples to be tested‐ΔCt of U6 in reference samples)]. The upstream primer sequences were designed based on the miRNA sequences obtained from the miRBase database (Release 20.0) as follows: miR-18a-5p forward primer 5′-ACACTCCAGCTGGGTAAGGTGCATCTAGTGCAGA-3′, miR-18a-5p reverse primer 5′-CTCAACTGGTGTCGTGGA-3′; miR-22-3p forward primer 5′-ACACTCCAGCTGGGAAGCTGCCAGTTGAAGAA-3′; miR-22-3p reverse primer 5′-CTCAACTGGTGTCGTGGA-3′.

### 2.7. Statistical Analysis

Normality of data distribution was assessed using the Kolmogorov-Smirnov test. The independent sample test, Chi-squared test, and nonparametric test were used to compare differences of demographic and clinical parameters between two groups. Spearman's correlation coefficient was used to test the correlation between miRNA markers, inflammatory cells to miRNA ratios, and cortisol and other clinical variables. The odds ratios (ORs) and their 95% confidence intervals (CIs) were calculated to assess the risk of miRNAs and inflammatory cells to miRNA ratios contributing to the presence of MetS using both univariate and binary logistic regression models with or without adjustment for covariates. ROC analysis was used to assess the biomarker potential of each miRNA and inflammatory cells to miRNA ratios for MetS, and AUC was used as the diagnostic index. The diagnostic performance of the miRNA and ratio panels was further evaluated using the predicted probability of being diagnosed with MetS as a surrogate marker to construct the ROC curve. A *P* value of less than 0.05 was considered statistically significant. The reported *P* values were two-tailed in all calculations. All statistical analyses were performed using SPSS 20.0.

## 3. Results

### 3.1. Basic Characteristics of the Study Subjects

The demographic and clinical characteristics of the study participants are presented in Table 1. Among the metabolic characteristics, most of them (including FG, FINS, HOMA-IR, TG, HDL-C, and SUA) have been shown significant differences between two groups, except for LDL-C. Most of the demographic parameters, including weight, height, BMI, WC, HC, WHR, SBP, and DBP, were significantly higher in the MetS group than those in the control group (*P* < 0.05). We also found that white blood cell (WBC), lymphocyte count (LC), and monocyte count (MC) were significantly higher in the MetS group than those in the control group, whereas hemoglobin was significantly lower in the MetS group than those in the control group, even after adjusting for gender. As for the hepatic and renal function, there were significant differences of alanine aminotransferase, glutamyl transpeptidase, serum creatinine, and estimated glomerular filtration rate in two groups. There was no significant difference in the distribution of smoking and alcohol consumption (*P* > 0.05). Compared with those controls, subjects with MetS were more likely to be physically inactive (*P* < 0.05).

### 3.2. Comparison of miRNA Levels and Stress Hormone between the Control and MetS Groups

**Figure 1:**
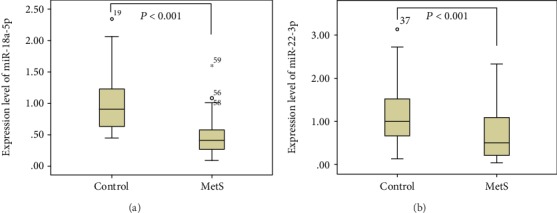
Comparison of microRNA expression in PBMCs among the MetS and control groups. Legend: the expression levels of miR-18a-5p in the MetS group and control group were 0.49 ± 0.31 and 1.05 ± 0.55 (median ± interquartile range), and *P* value was <0.001; the expression levels of miR-22-3p were 0.60 ± 0.44 and 1.18 ± 0.73 (median ± interquartile range), and *P* value was <0.001.

The expression levels of miR-18a-5p and miR-22-3p in the PBMCs and plasma levels of stress hormones including cortisol, IL-6, and TNF-*α* of the two groups are listed in Table 2. The expression levels of miR-18a-5p and miR-22-3p in MetS patients were significantly lower than those in control individuals (*P* < 0.001) (Figure 1). Levels of plasma cortisol and IL-6 in the MetS group were significantly higher than those in the control group (*P* = 0.003 and *P* = 0.006, respectively); however, there were no significant differences of plasma TNF-*α* levels in the two groups (*P* = 0.872).

### 3.3. Risk of miRNA Expression Contributed to the Presence of MetS

Univariate logistic regression analysis revealed that expression levels of miR-18a-5p and miR-22-3p were negatively associated with the presence of the MetS and control groups (*P* < 0.01) (Table 3). These associations were also confirmed in binary logistic regression analysis after adjusting for age, gender, and physical activity and further for WC. With a unit decrease of miR-18a-5p and miR-22-3p levels, there were 0.022 (95% CI: 0.003–0.152, *P* < 0.001) and 0.168 (95% CI: 0.063–0.453, *P* = 0.001) times of greater risks of MetS, respectively. These results indicated that miR-18a-5p and miR-22-3p are both independent protective factors for MetS.

### 3.4. Risk of the Inflammatory Cells to miR-18a-5p and miR-22-3p Ratios for MetS

Logistic regression analysis revealed that inflammatory cells to miR-18a-5p and miR-22-3p ratios were positively associated with the presence of MetS (*P* < 0.01) (Table 4). These associations were also confirmed in binary logistic regression analysis after adjusting for age, gender, and physical activity and further for WC. With an increase of inflammatory cells to miR-18a-5p and miR-22-3p ratios, there were 2.448 (95% CI: 1.608–3.727, *P* < 0.001)-, 1.314 (95% CI: 1.096–1.576, 0.003)-, 61.565 (95% CI: 9.084–417.226, <0.001)-, 3.393 (95% CI: 1.450–7.939, 0.005)-, 3.393 (95% CI: 1.450–7.939, <0.001)-, and 1.257 (95% CI: 1.079–1.464, 0.003)-folds of greater risks of MetS, respectively. Taken together, inflammatory cells to miR-18a-5p and miR-22-3p ratios were all independent risk factors for MetS.

### 3.5. The Correlation among miRNA Expression, Inflammatory Cells, Stress Hormones, and HOMA-IR

The Spearman correlation analysis showed that plasma cortisol was negatively associated with miR-18a-5p expression in the study subjects (Table 5), whereas there were no significant differences between miR-22-3p and cortisol. However, miR-22-3p was negatively correlated with IL-6 (*P* = 0.009) and TNF-*α* (*P* = 0.013) in the total subjects and negatively correlated with TNF-*α* (*P* = 0.017) in the control group. We also found that LC was positively correlated with cortisol and that LC and MC were negatively and positively associated with HOMA-IR, respectively.

As shown in Table 6, miR-18a-5p and miR-22-3p expressions had a moderate and negative correlation with various MetS components (including BMI, WC, HC, WH, SBP, DBP, plasma FG, plasma TG, and plasma UA) and positive correlation with plasma HDLC.

### 3.6. The Diagnostic Accuracy of the miRNAs

**Figure 2:**
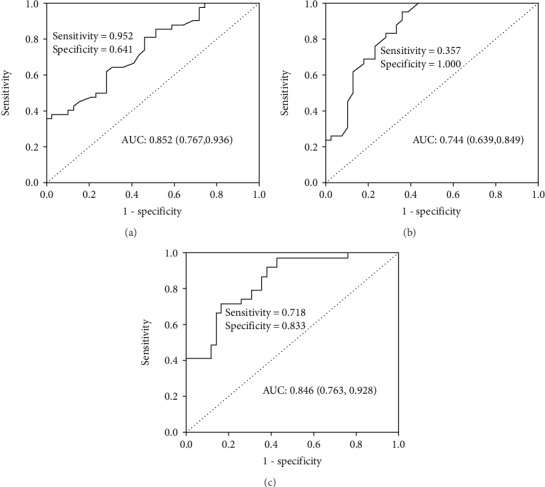
ROC curve analysis of miRNAs for MetS diagnosis. Legend: AUC estimation for the miRNAs: (a) miR-18a-5p, (b) miR-22-3p, and (c) ROC plot for the microRNA panel (miR-18a-5p, miR-22-3p) discriminating MetS. Abbreviations: AUC: area under the curve; ROC: receiver operating characteristic.

The diagnostic accuracy of miR-18a-5p and miR-22-3p, measured by AUC, was 0.852 (95% CI: 0.767-0.936) and 0.744 (95% CI: 0.639-0.849), respectively (*P* < 0.001). The corresponding sensitivity and specificity are presented in Figure 2. A stepwise logistic regression model to estimate the risk of being diagnosed with MetS was applied. The two miRNAs turned out to be significant predictors (*P* < 0.001). The predicted probability of being diagnosed with MetS from the logit model based on the two-miRNA panel, logit (*P* = MetS) = 3.020‐3.149∗miR − 18a − 5p–1.045∗miR − 22 − 3p, was used to construct the ROC curve. The AUC for the established miRNA panel was 0.846 (95% CI: 0.763-0.928, Figure 2(c)). These results reveal that miR-18a-5p and miR-22-3p are valuable biomarkers for differentiating MetS from healthy controls.

### 3.7. The Diagnostic Accuracy of the Inflammatory Cells to the Two miRNA Ratios

**Figure 3:**
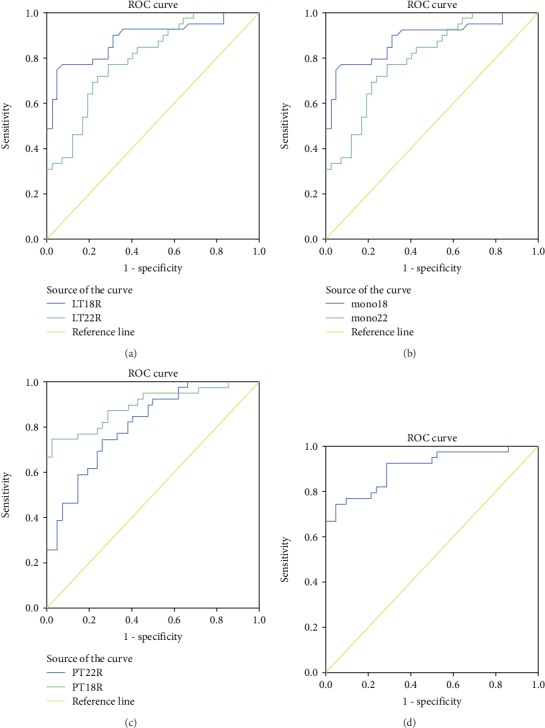
ROC curve analysis of inflammatory cells to the two miRNA ratios for MetS diagnosis. Legend: AUC estimation for the rates of inflammatory cells to the two miRNAs. (a) ROC curves for LT18R and LT22R, (b) ROC curves for MT18R and MT22R, (c) ROC curves for PT18R and PT22R, and (d) ROC curve for PT18R-PT22R panel. Abbreviations: AUC: area under the curve; ROC: receiver operating characteristic; LT18R: lymphocyte to miR-18a-5p ratio; LT22R: lymphocyte to miR-22-3p ratio; MT18R: monocyte to miR-18a-5p ratio; MT22R: monocyte to miR-22-3p ratio; PT18R: PBMCs to miR-18a-5p ratio; PT22R: PBMCs to miR-22-3p ratio.

The diagnostic accuracy of the lymphocyte to miR-18a-5p ratio (LT18R), lymphocyte to miR-22-3p ratio (LT22R), monocyte to miR-18a-5p ratio (MT18R), monocyte to miR-22-3p ratio (MT22R), PBMCs to miR-18a-5p ratio (PT18R), and PBMCs to miR-22-3p ratio (PT22R), measured by AUC, was 0.893 (95% CI: 0.820-0.965), 0.806 (95% CI: 0.714-0.899), 0.885 (95% CI: 0.809-0.961), 0.798 (95% CI: 0.823-0.966), 0.894 (95% CI: 0.823-0.966), and 0.809 (95% CI: 0.717-0.900), respectively (*P* < 0.001). The corresponding sensitivity and specificity were 74.4% and 97.6%, 74.4% and 73.8%, 76.9% and 92.9%, 76.9% and 71.4%, 74.4% and 97.6%, and 74.4% and 73.8%. The corresponding ROC curves are presented in Figure 3. A stepwise logistic regression model to estimate the risk of being diagnosed with MetS was applied. The two miRNAs turned out to be significant predictors (*P* < 0.001). The predicted probability of being diagnosed with MetS from the logit model based on the PT18R-PT22R panel, logit (*P* = MetS) = 0.721∗PT18R + 0.047∗PT22R‐3.686, was used to construct the ROC curve. The AUC for the established PMR panel was 0.905 (95% CI: 0.838-0.971, Figure 3(d)). These results reveal that the rates of inflammatory cells to miR-18a-5p and miR-22-3p are valuable biomarkers for differentiating MetS from healthy controls.

## 4. Discussion

The present study identified the relationship of expression levels of two stress-related miRNAs in PBMCs and the PBMCs to miR-18a-5p and miR-22-3p ratios between MetS patients and healthy controls in an occupational sample in Guangzhou. The decreased levels of miR-18a-5p and miR-22-3p were associated with the MetS presence, and they were both significant predictors for independence of MetS components. Besides, we have found that the expression levels of cortisol and IL-6 in MetS were significantly higher compared with healthy controls, and miR-18a-5p was negatively associated with cortisol. Our study also revealed that miR-18a-5p and miR-22-3p in PBMCs were potential markers for diagnosing MetS. But the main limitation of our study was the size of participants.

GR has two splice variants, named as GR*α* and GR*β*. GR*α* is the classical receptor which is bound with GCs to regulate gene expression, and GR*β* is served as an inhibitor of GR*α*, which can cause the GC resistance, providing a modest level of protection against the deleterious effects of GC exposure. Dexamethasone treatment and knockdown of GR*α* together with overexpression of GR*β* had opposite effects on glucose, amino acid, and fatty acid levels. The former treatment can increase glucose, amino acids, cholesterol, and fatty acids [15]. Another study has found that expression of GR*α* in myoblast under basal conditions had positive associations with levels of insulin resistance, BMI, percent body fat, and blood pressure, which may be involved in mechanisms contributing to the pathogenesis of the MetS [16]. But in another study, GR*α* mRNA was negatively correlated with BMI and triglycerides and reduced in obesity subjects in the abdominal subcutaneous and omental depots, which is controversial with the two former observations [17]. miR-18a and miR-22-3p can both inhibit the GR mRNA. In our study, we found that the expression levels of these two miRNAs decreased in the MetS group compared with the healthy group, which can be explained by the studies above.

The activation of the GR-responsive gene was strongly impaired by the overexpression of miR-18a. miR-18a is expressed widely throughout the body, which might cause the GC resistance. Inversely, decreased expression of miR-18a may induce the presence of metabolic dysfunction [10]. Previous study has found that expression levels of miR-18a in PBMCs were positively correlated with cortisol, CRH, and IL-6, and chronic stress increased plasma cortisol, which subsequently decreased mRNA expression of GR*α* and increased the GR*β*/GR*α* mRNA ratio. It has been also identified that the increased levels of miR-18a were associated with the T2DM presence [11]. Besides, it has been demonstrated that miR-18 could promote apoptosis of islet *β*-cells at least partially by inhibiting neuron navigator 1 expression and insulin production via suppression of the PI3K/AKT pathway [18]. In addition to the role in metabolic diseases, as an osteosarcoma-promoting miRNA, miR-18a-5p can also target the suppressor of cytokine signalling 5 in OS cells, which induced tumorigenesis [19]. Treated with chronic stress, the resilient rats (which were much less prone to the negative influence of stress than others) show a significantly decreased expression level of miR-18a-5p [20]. Our present study has found that the expression level of miR-18a-5p decreased in the MetS group, when compared with the control group, and was negatively associated with the stress hormone, cortisol. Based on our results and these earlier reports, the expression of miR-18a-5p is likely to be a protective factor of MetS.

miR-22-3p (also mentioned as miR-22), an abundantly expressed hepatic miRNA, is elevated in the diabetic mouse liver. miR-22-3p inhibited transcription factor 7 via binding to the 3′untranslated region, which increased the expression of enzymes of the gluconeogenic pathway in HepG2 cells. Inversely, miR-22-3p antagonism improved glucose tolerance and insulin sensitivity [21]. Interestingly, miR-22 was downregulated, and its target gene IL6 receptor (IL6R) was upregulated in both diabetic serum and glucose-induced INS-1E cells. Further study has identified that miR-22 overexpression or IL6R inhibition significantly strengthened cell survival and suppressed cell apoptosis; the potential mechanism was that miR-22 overexpression or IL6R inhibition could activate the phosphorylation of the JAK/STAT signalling pathway. High IL6 concentration inhibits insulin secretion. Through combining with the IL6R on the membrane of target cells, IL6 can induce a series of biological reactions in different target cells to participate in glucose and lipid metabolism [22]. Our present study also found that miR-22 was downregulated in MetS patients, and it was negatively associated with the IL6 level.

As a window, PBMCs (mainly consisting of lymphocytes) can convert psychosocial stress into cellular dysfunction and finally contribute to the pathophysiology of lifestyle-related diseases such as DM, CVD, and atherosclerosis [11]. Chen et al. have conducted a cross-sectional study on 852 participants (*n* = 598 with MetS and *n* = 254 without MetS) in south China and found that the severity of MetS was significantly positively associated with the WBC count, neutrophil count, and total lymphocyte count [23]. These results have been also discovered in our present study. We have identified that LT18R, LT22R, MT18R, MT22R, PT18R, and PT22R can be good indicators for diagnosing MetS. It is the first time that we investigated the relationship between the two miRNAs in PBMCs and the inflammatory cells to the two miRNA ratios and MetS at the population level. The reasonable diagnostic accuracy of miR-18a-5p, miR-22-3p, and the rates above indicates their clinical value in diagnosis of MetS. However, well-designed prospective studies with larger sample sizes are required to validate our findings. Besides, the expressions of these two miRNAs in circulation should be also detected, and the roles of these two miRNAs on GR and metabolic components need to be further identified.

## 5. Conclusion

Our findings suggest that miR-18a-5p and miR-22-3p in PBMCs may play a vital role in vulnerability to MetS by targeting GR. What is more, the inflammatory cells to the two miRNA ratios have demonstrated higher specificity and sensitivity in the diagnosis of MetS. These findings may unveil new targets for the prevention, diagnosis, and treatment of stress and inflammation-related disorders including MetS.

## Figures and Tables

**Table 1:** Demographic and clinical characteristics of study subjects.

Variable	MetS (*n* = 39)	Control (*n* = 42)	*P*	*P*′
Age (year)	43 ± 12	31.5 ± 10.75	<0.001^∗∗^	—
Gender (male/female)	31/8	19/23	0.001^∗∗∗^	—
Weight (kg)	78.06 ± 12.32	57.78 ± 8.31	<0.001^∗^	<0.001
Height (m)	1.68 ± 0.07	1.64 ± 0.07	0.030^∗^	0.028
BMI (kg/m)	27.77 ± 3.64	21.40 ± 2.47	<0.001^∗^	<0.001
WC (cm)	96.01 ± 8.94	76.30 ± 7.63	<0.001^∗^	0.001
HC (cm)	104.95 ± 6.67	92.51 ± 5.10	<0.001^∗^	<0.001
WHR	0.93 ± 0.05	0.82 ± 0.05	<0.001^∗^	<0.001
SBP (mmHg)	138 ± 15	113.5 ± 12.25	<0.001^∗∗^	<0.001
DBP (mmHg)	91 ± 9	69 ± 7.5	<0.001^∗∗^	0.013
FINS (*μ*IU/ml)	15.24 ± 7.13	7.12 ± 3.80	<0.001^∗∗^	<0.001
HOMA-IR	3.43 ± 2.76	1.52 ± 0.95	<0.001^∗∗^	0.002
FPG (mmol/l)	5.61 ± 0.85	4.79 ± 0.53	<0.001^∗∗^	<0.001
TG (mmol/l)	2.44 ± 1.59	0.81 ± 0.44	<0.001^∗∗^	<0.001
HDLC (mmol/l)	1.32 ± 0.34	1.89 ± 0.55	<0.001^∗^	<0.001
TC (mmol/l)	5.3 ± 1.72	4.41 ± 1.09	0.002^∗∗^	0.04
LDLC (mmol/l)	2.90 ± 1.24	2.53 ± 0.72	0.338^∗^	0.479
SUA (*μ*mol/l)	443.54 ± 104.60	309.83 ± 66.80	<0.001^∗^	<0.001
WBC (10^9^/l)	7.3 ± 3.1	5.7 ± 1.53	<0.001^∗∗^	0.001
Hb (g/l)	152.41 ± 13.18	141.83 ± 12.55	<0.001^∗^	0.013
LC (10^9^/l)	2.67 ± 0.7	1.88 ± 0.50	<0.001^∗∗^	<0.001
MC (10^9^/l)	0.49 ± 0.23	0.420.11	0.003^∗∗^	0.001
ALT (U/l)	26 ± 22	13 ± 9.25	<0.001^∗∗^	0.005
AST (U/l)	20 ± 14	18 ± 5	0.193^∗∗^	0.022
GGT (U/l)	33 ± 69	16 ± 6	<0.001^∗∗^	0.006
SCr (mg/dl)	87 ± 16	76.5 ± 26	0.011^∗∗^	0.974
eGFR (ml/(min·1.73 m^2^))	88.21 ± 16.51	96.50 ± 17.69	0.033^∗^	0.889
Smoking (*n*, %)	19, 48.70	16, 38.10	0.230^∗∗∗^	0.965
Alcohol use (*n*, %)	16, 41.00	14, 33.30	0.313^∗∗∗^	0.153
PA (*n*, %)	13, 33.33	28, 66.66	0.003^∗∗∗^	0.816
*Education*				
UU (*n*, %)	10, 25.60	7, 16.67	0.236^∗∗∗^	0.624
UA (*n*, %)	29, 74.40	35, 83.33		

Legend: ^∗^*P* value calculated with the independent sample test, and the data are expressed as the mean ± SEM. ^∗∗^*P* value calculated with the nonparametric test, and the data are expressed as the median ± interquartile range. ^∗∗∗^*P* value calculated with the *χ*^2^ test, and the data are expressed as the number and percentage. *P*′: *P* value calculated with binary logistic regression after adjusting for age and gender. Abbreviations: SUA: serum uric acid; WBC: white blood cell; Hb: hemoglobin; LC: lymphocyte count; MC: monocyte count; ALT: alanine aminotransferase; AST: aspartate transaminase; GGT: glutamyl transpeptidase; SCr: serum creatinine; eGFR: estimated glomerular filtration rate; PA: physical activity; UU: under university; UA: university and above; *n*: number.

**Table 2:** miRNA and hormonal characteristics in the two compared groups.

Variable	MetS (*n* = 39)	Control (*n* = 42)	*P*
miR-18a-5p	0.49 ± 0.31	1.05 ± 0.55	<0.001^∗∗^
miR-22-3p	0.60 ± 0.44	1.18 ± 0.73	<0.001^∗∗^
Cortisol (ng/ml)	455.98 ± 93.80	393.87 ± 86.34	0.003^∗^
IL-6 (pg/ml)	3.32 ± 3.7	2.04 ± 1.45	0.006^∗∗^
TNF-*α* (pg/ml)	5.87 ± 2.03	5.3 ± 1.97	0.872^∗^

Legend: ^∗^*P* value calculated with the independent sample test, and the data are expressed as the mean ± SEM. ^∗∗^*P* value calculated with the nonparametric test, and the data are expressed as the median ± interquartile range.

**Table 3:** The risk of miR-18a-5p and miR-22-3p for MetS.

Models	OR (95% CI)	*P* value
*miR-18a-5p*		
Univariate model	0.022 (0.003–0.152)	<0.001^∗^
Binary model 1	0.019 (0.002, 0.206)	0.001^∗∗^
Binary model 2	0.104 (0.008, 1.145)	0.092^∗∗∗^
*miR-22-3p*		
Univariate model	0.168 (0.063–0.453)	<0.001^∗^
Binary model 1	0.210 (0.068, 0.650)	0.007^∗∗^
Binary model 2	0.019 (0.000, 0.915)	0.045^∗∗∗^

Legend: this table shows the logistic regression analysis for the risk of miR-18a-5p and miR-22-3p for MetS. ^∗^*P* value calculated without adjustment. ^∗∗^*P* value adjusted for age, gender, and physical activity. ^∗∗∗^*P* value further adjusted for WC based on model 1. Abbreviations: OR: odds ratio; CI: confidence interval.

**Table 4:** The risk of inflammatory cells to miRNA ratios for MetS.

Models	OR (95% CI)	*P* value
*LT18R*		
Univariate model	2.5 (1.6–3.7)	<0.001^∗^
Binary model 1	3.8 (1.9, 7.7)	<0.001^∗∗^
Binary model 2	2.8 (1.0, 7.5)	0.049^∗∗∗^
*LT22R*		
Univariate model	1.3 (1.1–1.6)	0.003^∗^
Binary model 1	1.2 (1.0, 1.5)	0.03^∗∗^
Binary model 2	1.5 (1.0, 2.2)	0.093^∗∗∗^
*MT18R*		
Univariate model	61.6 (9.1–417.2)	<0.001^∗^
Binary model 1	279.3 (14.1, 5533.6)	<0.001^∗∗^
Binary model 2	510.6 (1.0, 279892.1)	0.053^∗∗∗^
*MT22R*		
Univariate model	3.4 (1.5–7.9)	0.005^∗^
Binary model 1	2.9 (1.2, 7.2)	0.022^∗∗^
Binary model 2	6.2 (0.5, 73.6)	0.151^∗∗∗^
*PT18R*		
Univariate model	2.1 (1.4–3.0)	<0.001^∗^
Binary model 1	3.1 (1.7, 5.6)	<0.001^∗∗^
Binary model 2	2.5 (1.0, 6.0)	0.047^∗∗∗^
*PT22R*		
Univariate model	1.3 (1.1–1.5)	0.003^∗^
Binary model 1	1.2 (1.0, 1.4)	0.03^∗∗^
Binary model 2^†^	1.4 (0.9, 2.0)	0.094^∗∗∗^

Legend: this table shows the logistic regression analysis for the risk of inflammatory cells to miRNA ratios for MetS. ^∗^*P* value calculated without any adjustment. ^∗∗^*P* value adjusted for age, gender, and physical activity. ^∗∗∗^Further adjustment for WC based on model 1. Abbreviations: LT18R: lymphocyte to miR-18a-5p ratio; LT22R: lymphocyte to miR-22-3p ratio; MT18R: monocyte to miR-18a-5p ratio; MT22R: monocyte to miR-22-3p ratio; PT18R: PBMCs to miR-18a-5p ratio; PT22R: PBMCs to miR-22-3p ratio; OR: odds ratio; CI: confidence interval.

**Table 5:** Spearman correlation among the miRNAs, inflammatory cells, stress hormones, and HOMA-IR.

	Cortisol	IL-6	TNF-*α*	HOMA-IR
*Total subjects*				
miR-18a-5p	-0.304^∗∗^	-0.073	-0.165	0.052
miR-22-3p	-0.139	-0.290^∗∗^	-0.367^∗^	0.199
LC	0.482^∗∗^	0.215	0.170	-0.576^∗∗^
MC	0.137	0.148	-0.074	0.269^∗^
*MetS group*				
miR-18a-5p	0.133	0.212	0.094	0.037
miR-22-3p	-0.124	-0.217	-0.195	0.141
LC	0.162	0.007	0.078	0.159
MC	-0.091	0.164	0.046	-0.004
*Control group*				
miR-18a-5p	-0.086	-0.075	0.008	-0.474^∗∗^
miR-22-3p	0.186	-0.127	-0.276^∗^	-0.246^∗^
LC	0.518^∗∗^	0.168	0.315	0.323^∗^
MC	0.198	-0.100	-0.195	0.084

Legend: ^∗^*P* < 0.05, ^∗∗^*P* < 0.01. Abbreviations: IL-6: interleukin-6; TNF-*α*: tumor necrosis factor-*α*, HOMA-IR: homoeostasis model assessment of insulin; LC: lymphocyte count; MC: monocyte count.

**Table 6:** Spearman correlation among the miRNAs and components of MetS.

	Total subjects		MetS group		Control group	
	miR-18a-5p	miR-22-3p	miR-18a-5p	miR-22-3p	miR-18a-5p	miR-22-3p
WC	-0.518^∗∗^	-0.381^∗∗^	-0.147	-0.097	0.191	-0.099
HC	-0.512^∗∗^	-0.261^∗^	-0.456^∗∗^	-0.068	0.329^∗^	0.161
WH	-0.526^∗∗^	-0.449^∗∗^	-0.196	-0.215	0.007	-0.258
BMI	-0.514^∗∗^	-0.279^∗^	-0.206	0.08	0.344^∗^	0.129
SBP	-0.502^∗∗^	-0.305^∗∗^	0.105	0.28	-0.067	-0.133
DBP	-0.462^∗∗^	-0.365^∗∗^	-0.004	-0.018	0.232	-0.047
FPG	-0.411^∗∗^	-0.283^∗^	-0.087	-0.02	0.065	-0.099
TG	-0.437^∗∗^	-0.327^∗∗^	-0.05	0.039	0.288	-0.051
HDLC	0.593^∗∗^	0.377^∗∗^	0.271	0.305	0.336^∗^	0.061
SUA	-0.612^∗∗^	-0.429^∗∗^	-0.265	-0.021	-0.429^∗∗^	-0.257

Legend: ^∗^*P* < 0.05, ^∗∗^*P* < 0.01. Abbreviations: WC: waist circumference; HC: hip circumference; WH: rate of waist circumference to hip circumference; BMI: body mass index; SBP: systolic blood pressure; DBP: diastolic blood pressure; FPG: fast plasma glucose; TG: triglyceride; HDLC: high-density lipoprotein cholesterol.

## Data Availability

The data used to support the findings of this study are available from the corresponding author upon request.
